# Fœtus in fœtu de siège rétropéritonéal

**DOI:** 10.11604/pamj.2019.34.78.15938

**Published:** 2019-10-10

**Authors:** Rim Hadhri, Ahlem Bellalah

**Affiliations:** 1 Service d'Anatomie et Cytologie Pathologiques, Hôpital Fattouma Bourguiba, Monastir, Tunisie

**Keywords:** Fœtus in fœtu, chirurgie, anatomie pathologique, Fetus in fetu, surgery, pathological anatomy

## Abstract

Fetus in Fetu (FIF) is an extremely rare congenital anomaly defined as a mass containing a vertebral axis often associated with other organs or limbs around this axis. We report the case of a female fetus aged 4 months presenting with retroperitoneal mass measuring 7x6x4cm, suggesting teratoma on computerized tomography (CT) scan. The mass was resected. Macroscopic examination showed fetiform mass covered by skin tissue and extending into upper and lower limb buds (A). Sectional views showed that it was centered by several osteocartilaginous fragments arranged in a linear fashion reminding the vertebral axis (B). Histological examination objectified the presence of glial tissue around a cerebral ventricle as well as skin, muscle and bone tissue. The diagnosis of FIF was retained. FIF is mainly reported at the level of the retroperitoneum followed by the sacro-caudal, intra-abdominal, cranial, buccal, mediastinal, pulmonary, renal and scrotal area. Diagnosis is made during the antenatal period in 15% of cases. The etiopathogenesis of FIF includes both the theory of monochorionic diamniotic monozygotic pregnancy in which an aberrant asymmetric twin becomes internalized in the other twin and the theory of a defective embryo implantation in the mesenchyme of its twin instead of the uterine wall. The differential diagnosis includes teratoma, meconium pseudocyst and ectopic pregnancy.

## Image en médecine

Le Fœtus in Fœtu (FIF) est une anomalie congénitale extrêmement rare définie comme une masse contenant un axe vertébral associé souvent à d'autres organes ou à des membres autour de cet axe. Nous rapportons l'observation d'un fœtus de sexe féminin âgé de 4 mois qui se présentait pour une masse rétropéritonéale mesurant 7x6x4cm évoquant un tératome à la tomodensitométrie. La masse était réséquée. L'examen macroscopique montrait une masse d'aspect fétiforme recouverte par un tissu cutané et se prolongeant par des ébauches de membres supérieurs et inférieurs (A). A la coupe, elle était centrée par plusieurs fragments ostéocartilagineux disposés d'une façon linéaire rappelant un axe vertébral (B). L'examen histologique objectivait la présence du tissu glial autour d'un ventricule cérébral en plus des tissus cutané, musculaire et osseux. Le diagnostic d'un FIF était retenu. Le FIF a été rapporté essentiellement au niveau du rétropéritoine suivi par les localisations sacro-coccygienne, intra-abdominale, crânienne, buccale, médiastinale, pulmonaire, rénale et scrotale. Le diagnostic est établi en anténatal dans 15% des cas. L'étiopathogénie du FIF inclut la théorie de grossesse monochoriale biamniotique monozygote par laquelle un aberrant jumeau asymétrique s'intériorise dans l'autre jumeau et la théorie d'implantation défectueuse d'un embryon dans le mésenchyme de son jumeau au lieu de la paroi utérine. Le diagnostic différentiel se pose avec le tératome, le pseudokyste méconial et la grossesse ectopique.

**Figure 1:**
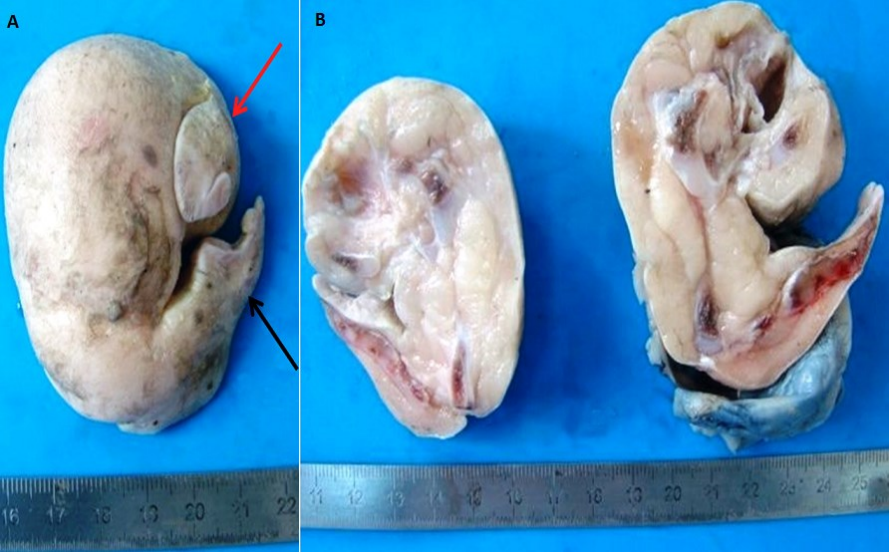
A) masse fétiforme se prolongeant par des ébauches de membres supérieurs (flèche rouge) et inférieurs (flèche noire); B) la masse est centrée par un axe vertébral

